# An Ultrasonic Contactless Sensor for Breathing Monitoring

**DOI:** 10.3390/s140815371

**Published:** 2014-08-20

**Authors:** Philippe Arlotto, Michel Grimaldi, Roomila Naeck, Jean-Marc Ginoux

**Affiliations:** 1 Laboratoire PROTEE EA3819, Université de Toulon, Avenue de l'Université, BP 20132, La Garde Cedex, France; E-Mail: grimaldi@univ-tln.fr; 2 Clinical Research Unit, Centre Hospitalier Intercommunal de Toulon La Seyne. 54, rue Henri Sainte Claire Deville, BP 1412, 83056, Toulon Cedex, France; E-Mail: roomila.naeck@gmail.com; 3 Laboratoire LSIS CNRS UMR 7296, Université de Toulon, Avenue de l'Université, BP 20132, La Garde Cedex, France; E-Mail: ginoux@univ-tln.fr

**Keywords:** breath monitoring, ultrasound, contactless, Doppler effect

## Abstract

The monitoring of human breathing activity during a long period has multiple fundamental applications in medicine. In breathing sleep disorders such as apnea, the diagnosis is based on events during which the person stops breathing for several periods during sleep. In polysomnography, the standard for sleep disordered breathing analysis, chest movement and airflow are used to monitor the respiratory activity. However, this method has serious drawbacks. Indeed, as the subject should sleep overnight in a laboratory and because of sensors being in direct contact with him, artifacts modifying sleep quality are often observed. This work investigates an analysis of the viability of an ultrasonic device to quantify the breathing activity, without contact and without any perception by the subject. Based on a low power ultrasonic active source and transducer, the device measures the frequency shift produced by the velocity difference between the exhaled air flow and the ambient environment, *i.e.*, the Doppler effect. After acquisition and digitization, a specific signal processing is applied to separate the effects of breath from those due to subject movements from the Doppler signal. The distance between the source and the sensor, about 50 cm, and the use of ultrasound frequency well above audible frequencies, 40 kHz, allow monitoring the breathing activity without any perception by the subject, and therefore without any modification of the sleep quality which is very important for sleep disorders diagnostic applications. This work is patented (patent pending 2013-7-31 number FR.13/57569).

## 1. Introduction

Breathing is one of the essential functions for the survival of most living beings. Many processes to measure the respiration rate have been proposed: using a stretch sensor or impedance meter to detect chest expansion [1–3], a pulse oximeter and extracting the respiration rate from the raw data [4], an accelerometer to detect chest expansion and contraction [5,6], measuring airflow pressure by oral or nasal cannula, and many others. In case of sleep apnea diagnosis for example, polysomnography (PSG), the commonly used test, employs nasal cannula and chest belts. The main problem common to all these techniques is the presence of a device directly in contact with the subject. As an example, for children this may induce rejection behavior leading them to remove the device. This is also true in the case of sleeping subjects, where the presence of the device can significantly disrupt the falling asleep and sleep. There have been several attempts to devise contactless methods. Electromagnetic waves can sense chest movement by Doppler effect [7–10] or by analyzing the signal backscattered by breathing movements [11] and ultrasound waves telemeters permits detection of small body displacements during respiration [12,13]. These solutions give good results but remain indirect because they do not analyze the true air flow and therefore cannot easily detect obstructive sleep apnea (OSA). Contactless direct air flow analysis is far less common in the literature. A microphone to detect exhalation sounds is proposed in [14], a high precision single point infrared sensor is use in [15] and a US patent [16] was granted for a method based on phase differences between two ultrasonic waves traveling in opposite directions. Nevertheless, the first method is perturbed by ambient noises and so the microphone needs to be placed close to the face of the subject to obtain good results. Concerning the second method, the sensor has to be fixed in an accurate position while the third method is complicated to implement because the two waves must travel exactly the same distance in absence of air flow.

This study proposes a new ultrasonic contactless device [17] to measure the presence or the absence of respiration and in the first case its relative intensity and rate. This sensor can be used alone in low cost systems or in conjunction with other remote sensors to increase reliability in sleep apnea diagnostic applications.

The physical principle of this measure is to “illuminate” the subject's head with an acoustic wave emitted by a transducer, then recovering and analyzing the reflected wave. Under these conditions, any movement, both in the subject himself and in the exhaled airflow, induce a frequency shift in the signal which is the Doppler effect. Every part of the head which participate to the reflection has approximately the same relative speed component in the direction of the receiver. Moreover, this speed is mostly low except in the case of very rapid movements. Hence, the surface of the skin returns a wave that remains nearly coherent and gives a single (or narrow) low Doppler frequency shift. In contrast the exhaled air flow returns a wave with a wide Doppler shift due to the presence of turbulence. The relative speeds are also higher than in the case of the movement which leads to a higher and broader Doppler shift. By frequency filtering and averaging, it therefore becomes possible to obtain and discriminate air flow signal and head and shoulder movement signal.

## 2. Experimental Section

The apparatus developed in the context of this work, allows monitoring the breathing of a subject lying on his back. These conditions correspond to the situation encountered in breath monitoring during sleep, like sleep apnea diagnosis. A 40 kHz ultrasound transmitter illuminates an area widely including the subject's head. One receiver, tuned to the same frequency, recovers the signal reflected from the scene. After reflection on the local environment, the incident wave is subject to various transformations related to physical and chemical characteristics of the media encountered, but also, to the movements of the neighboring objects. The result is a combination of a level attenuation, a frequency shift, (*i.e.*, Doppler effect), and a more complex effect resulting in a modification of spectral energy repartition *versus* time when the subject breathes out. The phenomenon, that we call “spectrum widening”, characterizes precisely the breath events and represents the core of this work.

**Figure 1.**
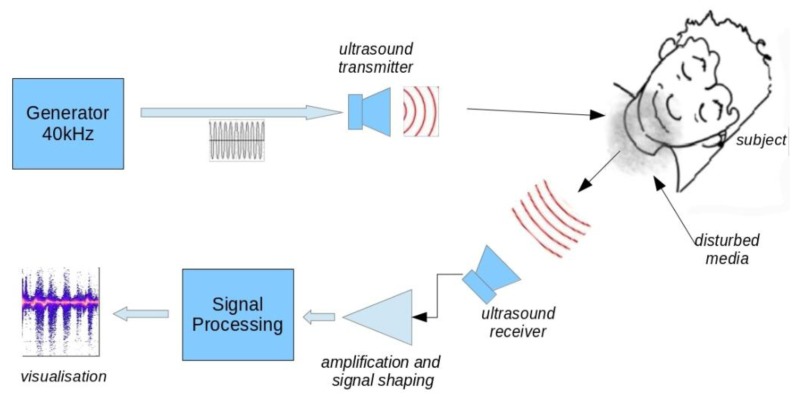
Block diagram of the device. The optimum distances and angles between the transmitter/receiver and the subject depend on the angle of the sensors relative to the head, the level of ultrasound source and the characteristics of the receiving system.

Figure 1 shows the block diagram of the device. We used inexpensive 40 kHz ultrasonic transducers with a 6 dB beamwidth of 55°. The emitted level was set to about 100 dB/0.0002 μbar. The transmitter was placed above patient head at a distance of 50 cm. We found that transmitter to head distance was not critical due to the ultrasound level and large beamwidth used. Receiver sensitivity is −65 dB *versus* 1 V/μbar. The receiver placement is a compromise between the signal strength and the need to allow the subject to move without hitting the sensor. The best signal is obtained when the receiver is close to the subject and directed to the nostrils. We used a distance of 30 cm for all data presented in this work. This distance could be increased with better receiver sensitivity and higher transmitted level but there is a physical limit where the exhaled air flow effect on the environment becomes insignificant.

### 2.1. Acquisition and Shaping of the Received Signal

**Figure 2.**
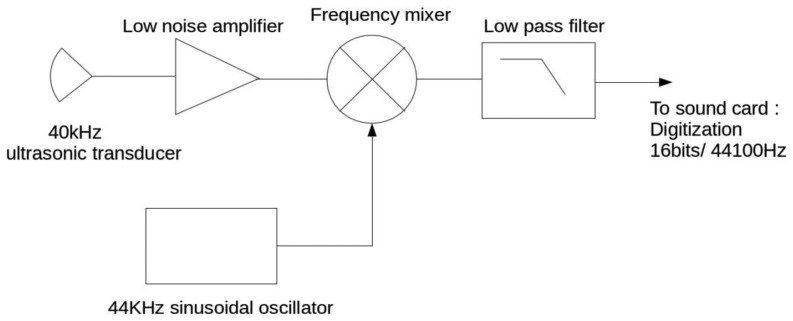
Block diagram of the receiver system.

The signal received by the ultrasonic receiver is centered around the source frequency, 40 kHz. To digitize that signal by respecting the Nyquist–Shannon sampling theorem, a high sampling frequency and a fast A/D converter would be necessary. As the received signal is narrow band, spanning about 2 kHz below and above 40 kHz, frequency translation to a much lower intermediate frequency is possible. We have chosen to translate it around 4 kHz using a frequency mixer. The received signal is mixed with a 44 kHz sinusoidal signal to generate 4 kHz and 84 kHz beat products. The high frequency product is removed with a low pass anti-aliasing filter before digitization. The block diagram is shown in Figure 2. This solution presents the technical advantage to allow a low frequency digitization as, for example, with an inexpensive computer sound card.

To visualize the spectral widening, we have used a time/frequency representation based on a short time Fourier transform (STFT) as:

(1) $$S\left(t^{\prime },\nu \right)=\int f\left(t\right)W\left(t-t^{\prime }\right)e^{2\pi i\nu t}\text{d}t$$

with *S*(*t′*,*v*) the STFT of the signal to be analyzed *f*(*t*) computed for each window centered at *t* = *t′*, *v* the frequency parameter, W(*t* − *t′*) the windowing function centered at *t* = *t′* and *i* stands for the imaginary unit satisfying *i*^2^ = −1.

For computation, a 44.1 kHz sampled signal is used with a Kaiser-Bessel windowing function [18] of size 65,536. This respects the Heisenberg inequality and allows a good compromise between time and frequency resolutions.

### 2.2. Physical Principles

**Figure 3.**
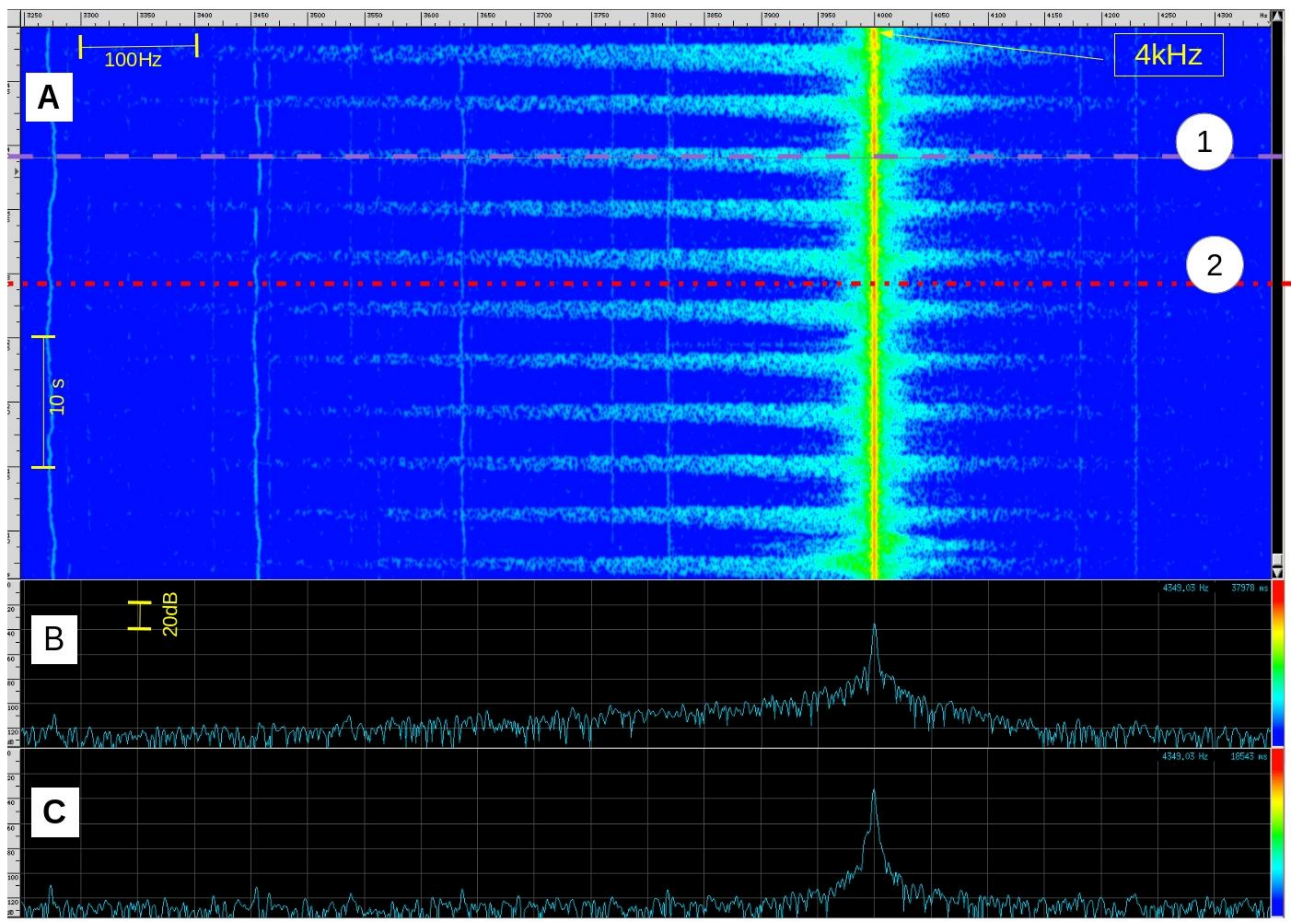
Signal visualization during rest and expiration phases. On top (**A**) a time (vertical) to frequency (horizontal) representation around the incident frequency pick. On bottom (**B**) the spectrum module during an expiration phase (1) and (**C**) the spectrum module during a rest phase (2), expressed in decibels.

The observed phenomenon (Figure 3) is the result of several combined physical effects whose principal is the Doppler effect. We can nevertheless separate two main parts: the overall effect generated by the mean speed of expired airflow (mean speed *v* ≈ 1 m·s^−1^ and Doppler frequency shift Δ*f* ≈ 100 Hz) and local effects caused by turbulence which produce greater frequency extensions (several hundreds of Hz above and/or below the transmit frequency).

The breathing exhalation flow can be assumed turbulent near its source (*i.e.*, nose or mouth). In such a flow, unsteady vortices appear on many scales and interact with each other. The friction between the exhaled flow and the static surrounding environment increases and interacts with the ultrasound wave.

### 2.3. Sound-Vorticity Interaction

The phenomenon of scattering of sound by vorticity is known since the 1950's. Obukhov [19] showed that an incident ultrasonic plane wave passing through an area with flow vorticity is scattered by it.

Inside a flow, we can show that any velocity field $\vec{u}$ can be decomposed into modes as:

(2) $$\vec{u}={\vec{u}}_{p}+{\vec{u}}_{v}+{\vec{u}}_{s}$$

with:

$$\begin{array}{c} \vec{\nabla }\cdot {\vec{u}}_{p}=0\;\text{and}\;\vec{\nabla }\wedge {\vec{u}}_{p}=\vec{0},\\ \vec{\nabla }\cdot {\vec{u}}_{s}\neq 0\;\text{and}\;\vec{\nabla }\wedge {\vec{u}}_{s}=\vec{0},\\ \vec{\nabla }\cdot {\vec{u}}_{v}=0\;\text{and}\;\vec{\nabla }\wedge {\vec{u}}_{v}=\vec{\Omega }\left(\vec{r},t\right)\neq \vec{0}, \end{array}$$

where *u⃗_p_* is a potential flow, *u⃗_s_* identify a sound wave and Ω⃗(*r⃗*,*t*) the vorticity.

**Figure 4.**
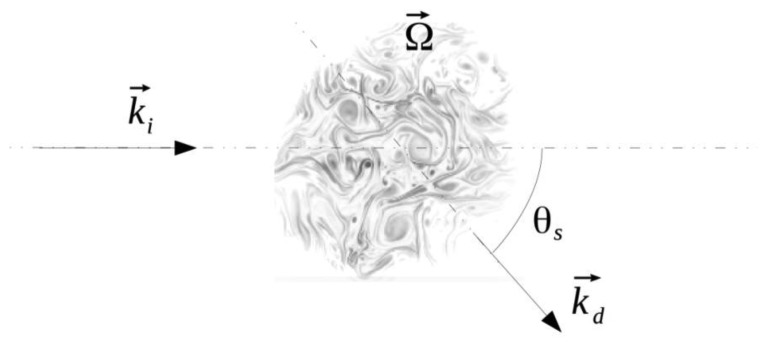
Observation angle of the scattered wave.

Chu [20], in 1958, showed the existence of a coupling between these different modes. More recently, Lund and Rojas [21] have derived a convenient expression that connects the acoustic scattered pressure, *p_scat_* directly to the Fourier transform of the vorticity field:

(3) $$p_{\text{scat}}\left(\vec{r},\nu \right)=p_{\text{inc}}\frac{-\cos\left(\theta_{s}\right)\sin\left(\theta_{s}\right)}{1-\cos\left(\theta_{s}\right)}\frac{2i\pi^{3}\nu }{c^{2}}\frac{e^{i{\vec{k}}_{d}\cdot \vec{r}}}{r}\Omega \left(\vec{q},\nu -\nu_{0}\right)$$

with:

*p_inc_* the pressure amplitude of the plane incident wave of frequency *v*_0_ and wave vector ${\vec{k}}_{i}=\frac{2\pi \nu_{0}}{c}\hat{i}$,
θ*_s_* the diffusion angle (Figure 4),
*v* the scattered wave frequency of wave vector ${\vec{k}}_{d}=\frac{2\pi \nu }{c}\hat{d}$,
$\vec{q}={\vec{k}}_{d}-{\vec{k}}_{i}$ the scattered wave vector,
Ω the vorticity component perpendicularly to the plane.

This shows a linear relationship between the acoustic scattered pressure *p_scat_*, the incident pressure *p_inc_* and the wave frequency *v*, thus the system would benefit from increasing the transmit level and the wave frequency. However, practical transducers of high frequency tend to have a reduced beamwidth which could limit the ability to detect breathing for all head positions.

The angular term of the Lund formula that modulates the acoustic scattering intensity will be denoted by:

(4) $$L\left(\theta_{s}\right)=\frac{-\cos\left(\theta_{s}\right)\sin\left(\theta_{s}\right)}{1-\cos\left(\theta_{s}\right)}$$

**Figure 5.**
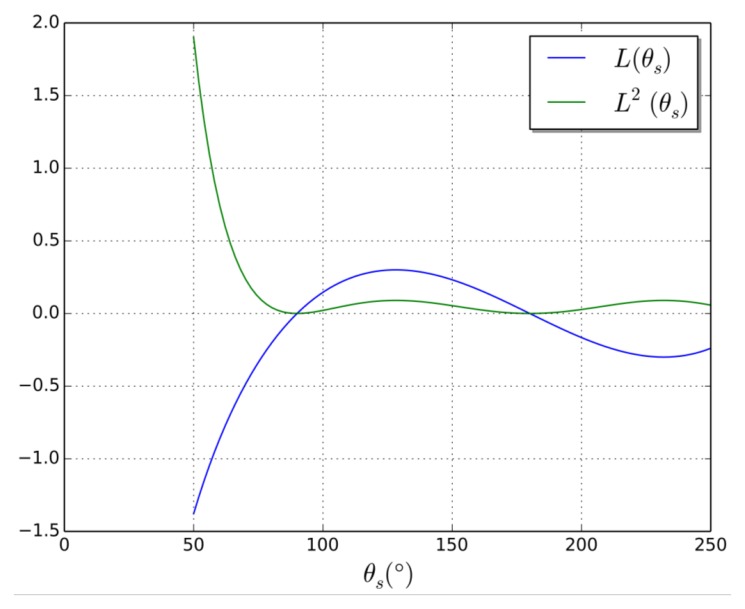
Observation angle of the scattered wave. We can see that there is no scattered signal θ*_s_* = 90° or backscattered signal θ*_s_* = 180° with a local maximum for θ*_s_* = 130°.

Figure 5, shows the evolution of *L*(θ*_s_*) and *L*^2^(θ*_s_*). This latter term modulates the acoustic scattering intensity. Note that the apparent divergence of this expression for small scattering angles is an artifact due to the conditions of validity of the Lund formula. Low angles would allow better scattered signal, but are not practical in our application. The optimum angle, experimentally determined, is in accordance with the second local maximum.

This shows that it is theoretically possible to measure the vorticity of a flow by diffusion of ultrasounds that corresponds to what is done by interpreting the spectrum of the acoustic received signal.

More physically, when the wave excites a vortex, this latter is advected by the oscillating velocity field of small amplitude *u⃗_p_*, the sound wave, and begins to oscillate. As an unsteady vortex is a sound source, the oscillating vortex will emit sound. Thus, the excitation by the incident wave will be scattered by the vorticity, in the same way that light is scattered by matter.

### 2.4. Influence of Experimental Conditions

- Oral or Nasal Breathing

**Figure 6.**
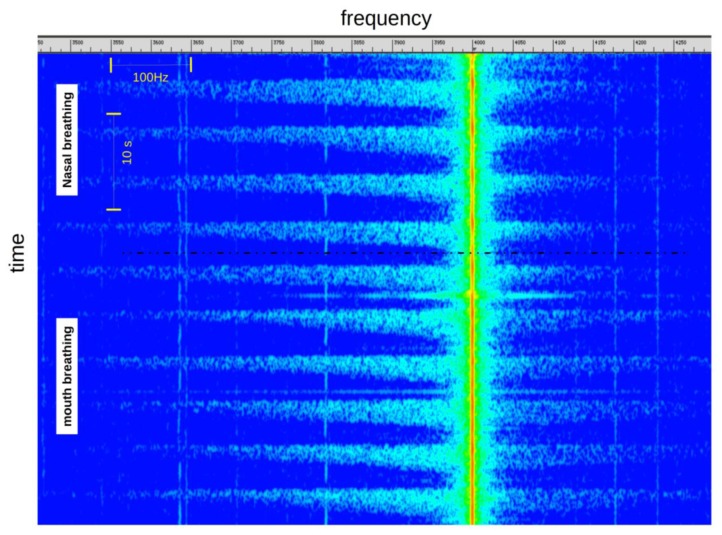
Signal visualization during nasal and mouth breathing.

Typically, the phenomenon is similar whatever the type of breathing (nasal or oral). We can just observe a slightly lower level of the signal in case of nasal breathing (Figure 6).

- Position of the Subject Head, Relative to the Sensor

**Figure 7.**
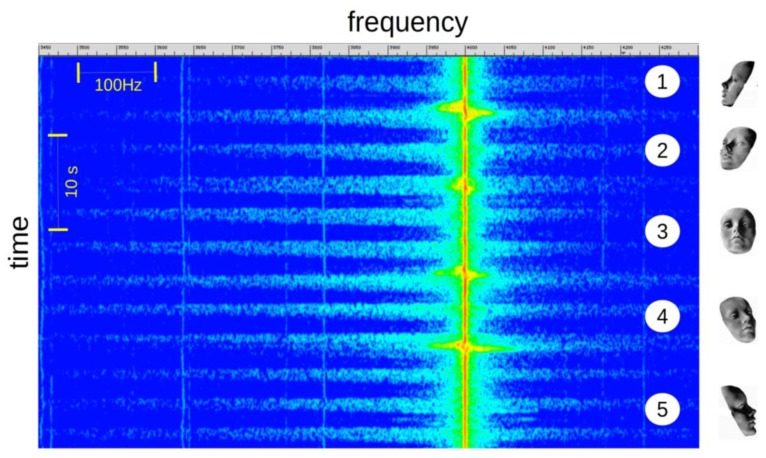
Signal visualization *versus* head position. On the right, the face, as viewed from the sensor.

The Figure 7 shows the change in the signal intensity according of the inclination of the subject's head. The signal level is maximum in front of the sensor (3) and gradually decreases with the angle of incidence. This level variation is not a problem for the application of apnea detection as the signal to noise ratio is acceptable for all positions. We can observe a high level Doppler offset of the incident wave frequency when the subject changes his position.

### 2.5. Influence of Subject or Surrounding Movements

**Figure 8.**
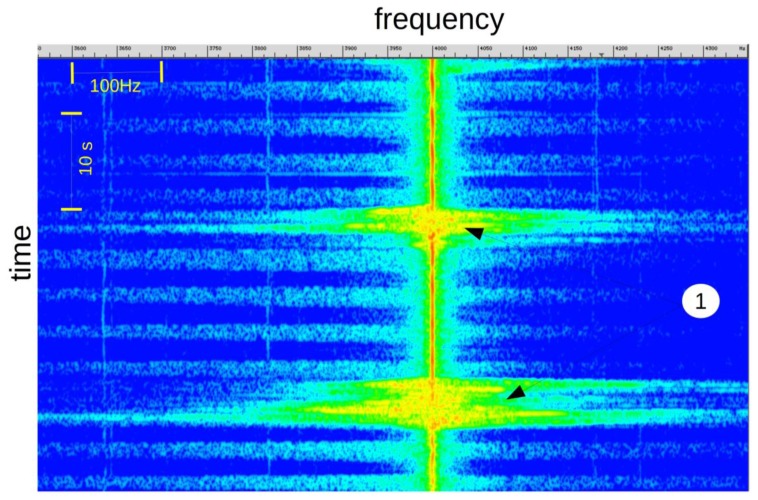
Signal visualization during large and rapid subject movements.

During rapid subject movements (Figure 8, mark 1), like sleeping position changes, the breathing signal is embedded in the Doppler shift. In this situation, it is difficult to verify the presence, or absence, of breathing activity.

### 2.6. Obtaining a Breathing Signal

In order to characterize respiration, we need a scalar signal from which breathing intensity and respiratory rate can be calculated.

**Figure 9.**
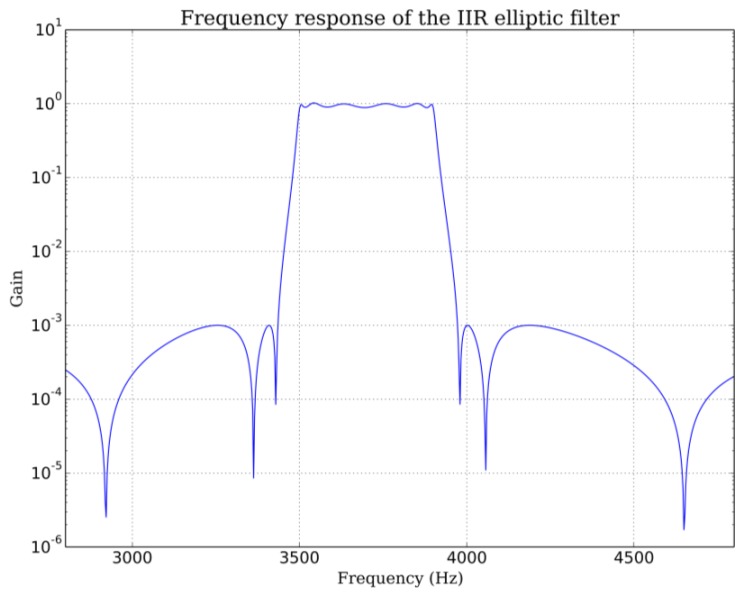
Frequency response of the IIR elliptic filter.

The received signal is first bandpass filtered by an infinite impulse filter (IIR). The bandpass is chosen to avoid the noisy “movement region” near the transmit frequency. One can use either the low or high frequency “breathing zone” or both. Breathing signal intensity on these two zones depends on sensor orientation relative to the subject. In our experimental configuration, signal strength is greater on the low frequency side (Figure 3). For a 4 kHz carrier received after down-mixing, we use a 3500–3900 Hz bandpass elliptic IIR filter with 60 dB stop band attenuation and 1 dB bandpass ripple (Figure 9). To further improve rejection, we apply this filter twice. The 3900 Hz highpass frequency is chosen to exclude the frequency components close to the carrier frequency because the signal level in this zone varies with head position and head movements. The choice of the lower frequency is a compromise. On very deep exhalations, spectral widening can extent down to 3000 Hz but average exhalations do not produce significant signal down to 3500 Hz. Lowering the lowpass frequency further than 3500 Hz would not add more information and would introduce unwanted noise on average exhalations. This would decrease the average signal to noise ratio.

Adult respiratory rate can vary from 0.2 to 0.5 Hz, children and newborn can show rate up to 1 Hz. Thus we choose a 10 Hz sample rate for our breathing signal. The root mean square (RMS) value of the bandpass filtered signal is computed every 100 ms (1/10 Hz). This RMS signal is low-pass filtered with a Butterworth filter to give the breathing signal. The optimal low pass frequency was determined by the residual analysis method [22]. The residual *Res*, defined mathematically as the root mean square value of the difference between filtered and unfiltered data, was plot over a set of 100 cutoff frequencies *f_c_* evenly spaced from 0.1 Hz up to the Nyquist frequency (5 Hz for 100 ms sample time in our case):

(5) $$Res\left(f_{c}\right)=\sqrt{\frac{1}{N_{s}}{\sum_{i=0}^{N}\left(x_{i}-{x^{\prime }}_{i}\right)}^{2}}$$

where:

*N_s_* is the number of samples,
*x_i_* is the *i*th unfiltered RMS value,
$x_{i}^{'}$ is the *i*th filtered RMS value.

**Figure 10.**
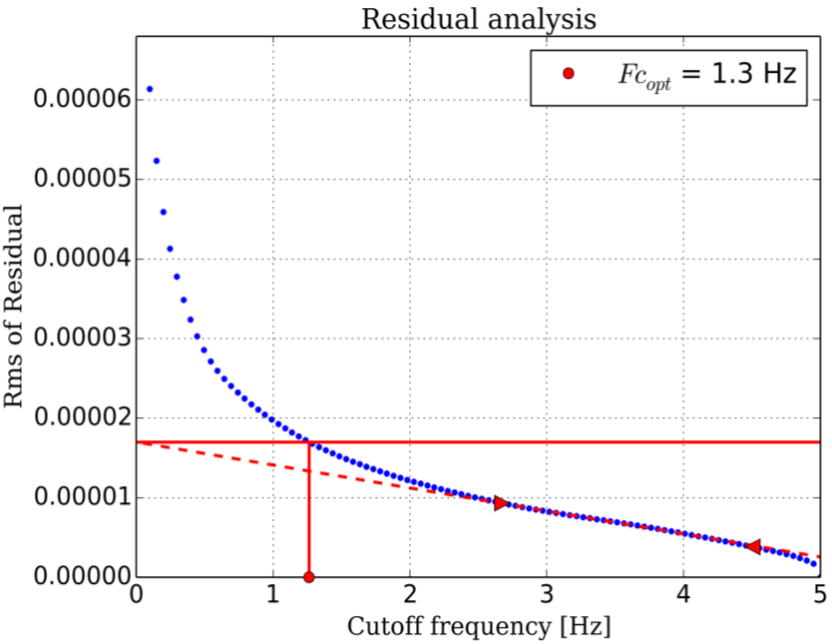
Residual analysis: plot of the residual value of typical breathing data for different cutoff frequencies. The optimal cutoff is chosen at the point where noise contribution equals signal contribution.

**Figure 11.**
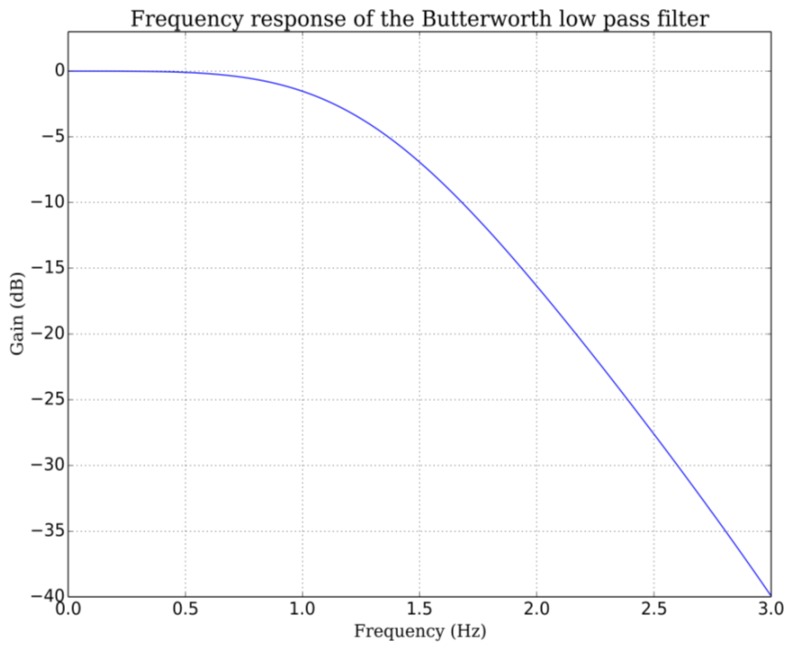
Frequency response of the Butterworth lowpass filter used for the breathing signal.

**Figure 12.**
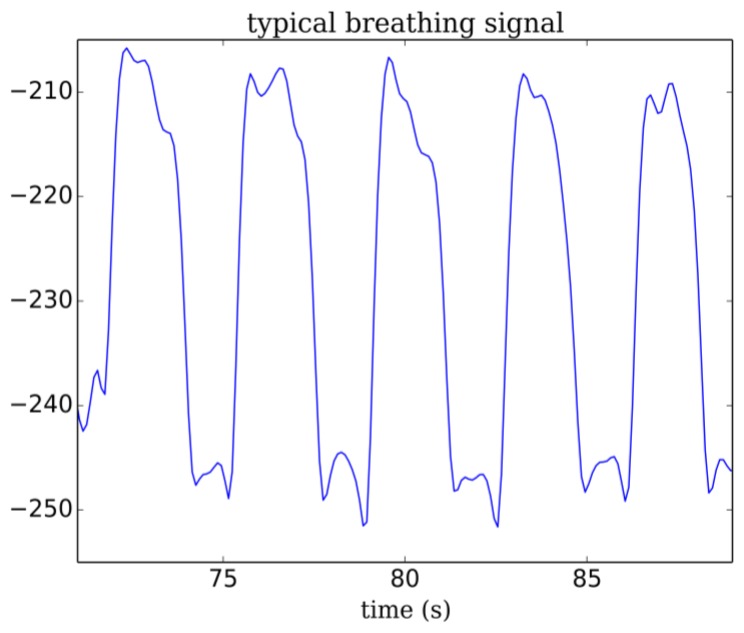
Typical breathing signal *versus* time (s). Arbitrary unit.

If we make the assumption that data is contaminated by a white noise, the residual would vary linearly with the cutoff frequency. This is the case for our breathing data above 2.5 Hz and up to 4.5 Hz. If we extrapolate this linear region up to the point it intercepts the y-axis, we can estimate the RMS value of the noise (dash red line in Figure 10). The optimal frequency is chosen at the point where noise contribution to the residual equals signal contribution. This is a compromise between the amount of noise allowed to pass through the filter and the signal distortion. This analysis, repeated other a range of breathing data obtained from adults at rest leads to optimal cutoffs between 1.2 to 1.5 Hz. The lowpass frequency was set to 1.5 Hz for all data presented in this work (Figure 11). A typical breathing signal is shown in Figure 12.

### 2.7. Obtaining a Signal Representing the Subject Motion

The breathing signal is disturbed in presence of large and rapid movements because the received spectrum is also widened during such events. Little head or shoulder motions as they occurred during sleep have no effect on the breathing signal but when the patient changes sleeping position or moves his arms, the breathing signal should not be taken into account. Thus we need a movement signal to detect situations where the breathing signal is too perturbed to give meaningful information.

**Figure 13.**
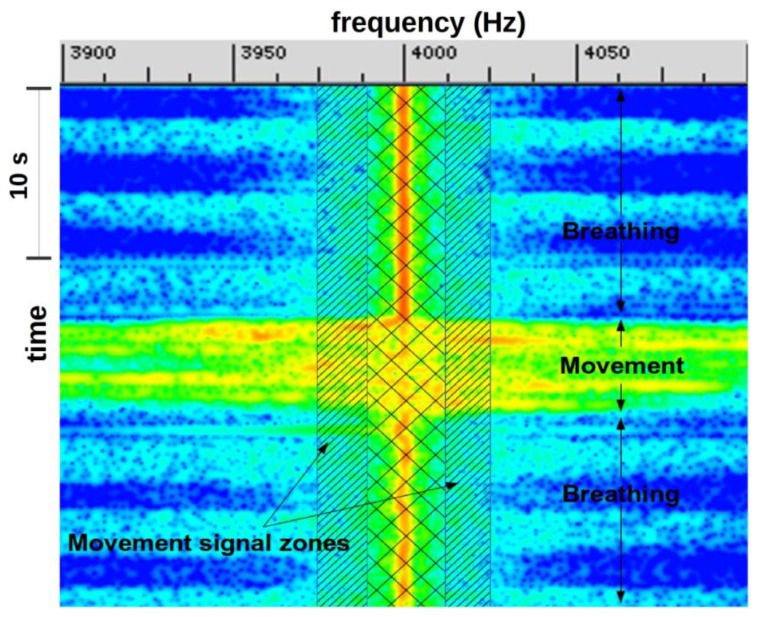
Breathing and movement combination. Rapid motions widen the spectrum and impede breathing detection. The single hatched area corresponds to the frequencies from where the movement signal is computed.

**Figure 14.**
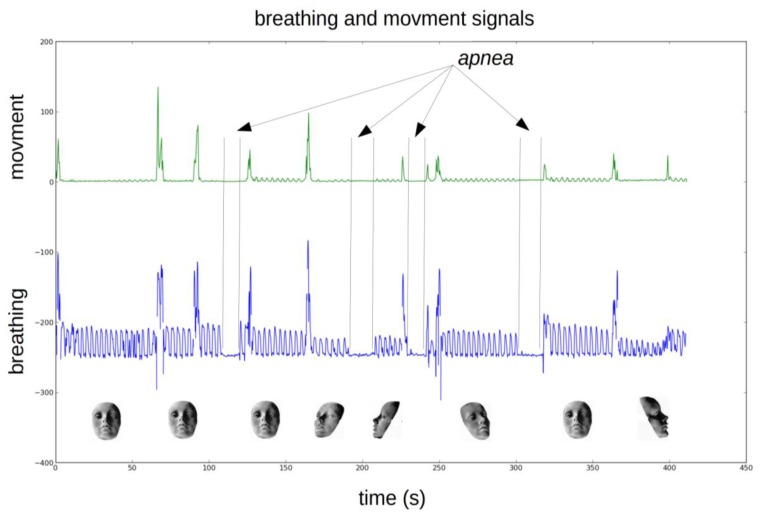
Breathing and movement signals. The movement signal (green; linear scale) increases when the patient changes position. The breathing signal (blue) shows expirations and four apneas. When the movement signal increases the breathing signal is meaningless.

We derived a movement signal by tracking energy variation (using standard deviation) in a very narrow window below and above the center frequency. This was done by computing the Fourier transform of the received signal other a 371 ms time slice (16,384 samples at 44,100 Hz) and keeping only bins of interest. Figure 13 shows the two movement zones of approximately 12 Hz apart the center frequency from where the signal movement is computed. A 25 Hz zone centered on the center frequency was excluded because the signal value in this area can vary widely due to patient position. Figure 14 shows a movement signal and a breathing signal. The movement signal increases when the patient changes position. When the movement signal increases the breathing signal is meaningless.

## 3. Results and Discussion

Several validations were performed with our sensor to determine its performance. A first one, qualitative, consisted of positioning a breathing subject under the sensor and observing in real time the spectrogram (time/frequency representation). Under these conditions, we can verify the perfect correspondence between the spectrogram and the breathing activity during normal breathing, simulated apnea or movements.

**Figure 15.**
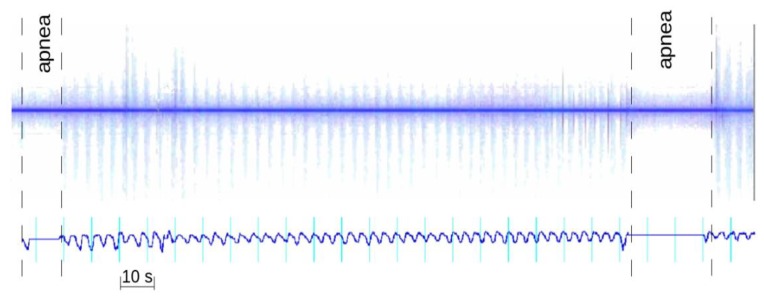
Correspondence between the received spectrum and a nasal cannula signal.

A second validation, more quantitative, was to equip the subject with a polygraph currently used in sleep laboratories to diagnose sleep apnea syndrome (Cid102L, Cidelec^®^, Sainte Gemmes sur Loire, France). It consists in a pressure sensor connected to a nasal cannula and to record both signals over a period of about 1 h which corresponds to about 1000 breathing cycles (Figure 15). During this period, the subject breathes through the mouth, the nose and simulates apnea and performs movements compatible with sleep. This experience showed the synchronization of the pressure signal provided by the nasal cannula with our signal spectrogram. However, the presence of the cannula in the nostrils derives a significant part of the exhaled air flow and therefore reduces the sensitivity of our device.

## 4. Conclusion

The sensor presented in this work represents a good solution for contactless breathing monitoring. Its applications span from real-time monitoring to sleep apnea diagnosis. Its contactless feature makes it suitable for breathing monitoring of infants or premature, and more generally monitoring of subjects at risk of having respiratory distress.

In future works, we plan to use several receivers evenly spaced around the subject head to allow us to remove the angular dependence of the breathing signal. Using a Fleish pneumotachograph we should be able to calibrate our system in order to obtain absolute measurements. In a second experiment, we will try a different signal processing methodology based on pattern matching classification of the different breathing states. To do this, and complete this study, a large measurement campaign on real patients is planned in collaboration with the CHITS (Toulon Hospital).
